# *cis*-Aqua­bis­(2,2′-bipyrimidine-κ^2^ *N* ^1^,*N* ^1′^)iodidomanganese(II) iodide monohydrate

**DOI:** 10.1107/S160053681103875X

**Published:** 2011-09-30

**Authors:** Kwang Ha

**Affiliations:** a School of Applied Chemical Engineering, The Research Institute of Catalysis, Chonnam National University, Gwangju 500-757, Republic of Korea

## Abstract

The asymmetric unit of the title compound, [MnI(C_8_H_6_N_4_)_2_(H_2_O)]I·H_2_O, consists of a cationic Mn^II^ complex, an I^−^ anion and a solvent water mol­ecule. In the complex, the Mn^II^ ion is six-coordinated in a distorted octa­hedral environment defined by four N atoms of the two chelating 2,2′-bipyrimidine (bpym) ligands, one I^−^ anion and one O atom of a water ligand. The dihedral angle between the least-squares planes of the two bpym ligands [maximum deviation = 0.092 (7) Å] is 79.9 (1)°. In the crystal, the complex, anion and solvent water mol­ecule are linked by inter­molecular O—H⋯O, O—H⋯I and O—H⋯N hydrogen bonds.

## Related literature

For the crystal structures of mononuclear 2,2′-bipyrimidine Mn^II^ complexes, see: Hong *et al.* (1996 ▶); Smith *et al.* (2001 ▶); Ha (2011 ▶).
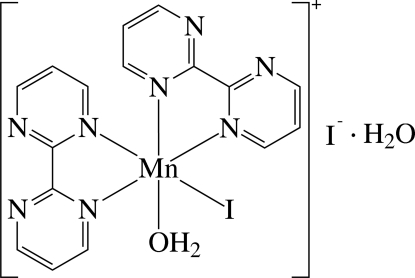


## Experimental

### 

#### Crystal data

[MnI(C_8_H_6_N_4_)_2_(H_2_O)]I·H_2_O
*M* *_r_* = 661.11
Triclinic, 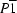

*a* = 7.8799 (9) Å
*b* = 12.8197 (15) Å
*c* = 12.9563 (15) Å
α = 113.302 (2)°
β = 101.695 (2)°
γ = 104.053 (3)°
*V* = 1098.6 (2) Å^3^
*Z* = 2
Mo *K*α radiation
μ = 3.44 mm^−1^
*T* = 200 K
0.18 × 0.17 × 0.07 mm

#### Data collection

Bruker SMART 1000 CCD diffractometer
Absorption correction: multi-scan (*SADABS*; Bruker, 2000 ▶) *T*_min_ = 0.859, *T*_max_ = 1.000
8099 measured reflections
5309 independent reflections
3106 reflections with *I* > 2σ(*I*)
*R*_int_ = 0.035

#### Refinement

*R*[*F* ^2^ > 2σ(*F* ^2^)] = 0.052
*wR*(*F* ^2^) = 0.126
*S* = 1.07
5309 reflections
262 parameters
H-atom parameters constrained
Δρ_max_ = 1.69 e Å^−3^
Δρ_min_ = −1.98 e Å^−3^

### 

Data collection: *SMART* (Bruker, 2000 ▶); cell refinement: *SAINT* (Bruker, 2000 ▶); data reduction: *SAINT*; program(s) used to solve structure: *SHELXS97* (Sheldrick, 2008 ▶); program(s) used to refine structure: *SHELXL97* (Sheldrick, 2008 ▶); molecular graphics: *ORTEP-3* (Farrugia, 1997 ▶) and *PLATON* (Spek, 2009 ▶); software used to prepare material for publication: *SHELXTL* (Sheldrick, 2008 ▶).

## Supplementary Material

Crystal structure: contains datablock(s) I. DOI: 10.1107/S160053681103875X/bv2192sup1.cif

Structure factors: contains datablock(s) I. DOI: 10.1107/S160053681103875X/bv2192Isup2.hkl

Additional supplementary materials:  crystallographic information; 3D view; checkCIF report

## Figures and Tables

**Table:** 

Mn1—O1	2.115 (5)
Mn1—N1	2.256 (6)
Mn1—N4	2.262 (6)
Mn1—N5	2.270 (6)
Mn1—N8	2.304 (6)
Mn1—I1	2.8048 (13)

**Table:** 

N1—Mn1—N4	72.8 (2)
N5—Mn1—N8	72.2 (2)

**Table 2:** Hydrogen-bond geometry (Å, °)

*D*—H⋯*A*	*D*—H	H⋯*A*	*D*⋯*A*	*D*—H⋯*A*
O1—H1*A*⋯O2^i^	0.84	1.93	2.767 (8)	172
O1—H1*B*⋯O2	0.84	1.82	2.645 (8)	167
O2—H2*A*⋯I2	0.84	2.60	3.423 (6)	168
O2—H2*B*⋯N6^ii^	0.84	2.06	2.871 (8)	162
O2—H2*B*⋯N7^ii^	0.84	2.38	2.918 (9)	122

Symmetry codes: (i) 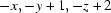; (ii) 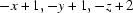.

**Fig. 1.**
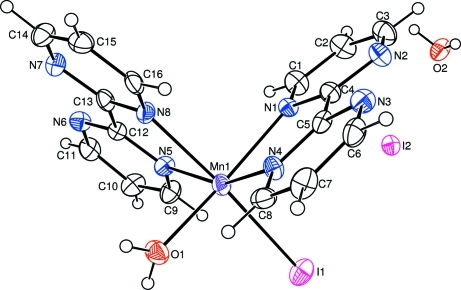
The structure of the title compound, with displacement ellipsoids drawn at the 40% probability level for non-H atoms.

**Fig. 2.**
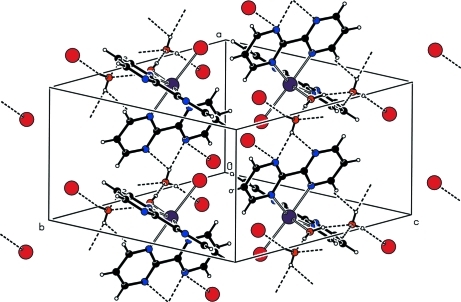
View of the unit-cell contents of the title compound. Hydrogen-bond interactions are drawn with dashed lines.

## supplementary crystallographic information

### Comment

Mononuclear Mn^II^ complexes of the 2,2'-bipyrimidine (bpym; C_8_H_6_N_4_) ligand, such as [Mn(bpym)_2_(H_2_O)_2_](ClO_4_)_2_.2H_2_O (Hong *et al.*, 1996), [Mn(bpym)_2_(H_2_O)_2_](BF_4_)_2_.2H_2_O (Smith *et al.*, 2001) and [MnBr_2_(bpym)_2_].CH_3_NO_2_ (Ha, 2011), have been investigated previously.

The asymmetric unit of the title compound, [MnI(bpym)_2_(H_2_O)]I.H_2_O, consists of a cationic Mn^II^ complex, an I^-^ anion and a solvent water molecule (Fig. 1). In the complex, the Mn^II^ ion is six-coordinated in a distorted octahedral environment defined by four N atoms of the two chelating bpym ligands, one I^-^ anion and one O atom of a water ligand in a *cis*-N_4_IO coordination geometry. The small bite of the bpym ligand results in N—Mn—N chelating angles of 72.2 (2)° and 72.8 (2)° and (Table 1) contributes to the distortion of the octahedron, which results in non-linear *trans* angles (O1—Mn1—N1 = 166.1 (2)°, I1—Mn1—N8 = 174.35 (16)° and N4—Mn1—N5 = 159.3 (2)°). The Mn—N(bpym) bond lengths are slightly different and longer than the Mn—O(H_2_O) bond. Because of the different *trans* effects of the I and O atoms, the Mn1—N8 bond *trans* to the I atom is somewhat longer than the Mn1—N1 bond *trans* to the O atom. The dihedral angle between the least-squares planes of the two bpym ligands [maximum deviation = 0.092 (7) Å] is 79.9 (1)°. In the crystal structure, the complex, anion and solvent water molecule are linked by intermolecular O—H···O, O—H···I and O—H···N hydrogen bonds (Fig. 2, Table 2). In addition, the complexes display numerous inter- and intramolecular π-π interactions between adjacent pyrimidine rings, the shortest ring centroid-centroid distance being 3.648 (5) Å.

### Experimental

To a solution of 2,2'-bipyrimidine (0.1587 g, 1.003 mmol) in acetone (40 ml) was added MnI_2_ (0.1540 g, 0.499 mmol) and refluxed for 3 h. The formed precipitate was separated by filtration, washed with acetone and dried at 50°C, to give a yellow powder (0.0701 g). Crystals suitable for X-ray analysis were obtained by slow evaporation from a 2-butanone solution.

### Refinement

Carbon-bound H atoms were positioned geometrically and allowed to ride on their respective parent atoms [C—H = 0.95 Å and *U*_iso_(H) = 1.2*U*_eq_(C)]. The H atoms of the water ligand and solvent molecule were located from Fourier difference maps then allowed to ride on their parent O atoms in the final cycles of refinement with O—H = 0.84 Å and *U*_iso_(H) = 1.5 *U*_eq_(O). The highest peak (1.69 e Å^-3^) and the deepest hole (-1.98 e Å^-3^) in the difference Fourier map are located 1.15 Å and 0.85 Å from the atoms I2 and I1, respectively.

### Crystal data

**Table:**

[MnI(C_8_H_6_N_4_)_2_(H_2_O)]I·H_2_O	*Z* = 2
*M**_r_* = 661.11	*F*(000) = 630
Triclinic, *P*1	*D*_x_ = 1.999 Mg m^−^^3^
Hall symbol: -P 1	Mo *K*α radiation, λ = 0.71073 Å
*a* = 7.8799 (9) Å	Cell parameters from 2443 reflections
*b* = 12.8197 (15) Å	θ = 2.8–27.6°
*c* = 12.9563 (15) Å	µ = 3.44 mm^−^^1^
α = 113.302 (2)°	*T* = 200 K
β = 101.695 (2)°	Plate, yellow
γ = 104.053 (3)°	0.18 × 0.17 × 0.07 mm
*V* = 1098.6 (2) Å^3^	

### Data collection

**Table:**

Bruker SMART 1000 CCD diffractometer	5309 independent reflections
Radiation source: fine-focus sealed tube	3106 reflections with *I* > 2σ(*I*)
graphite	*R*_int_ = 0.035
φ and ω scans	θ_max_ = 28.3°, θ_min_ = 1.8°
Absorption correction: multi-scan (*SADABS*; Bruker, 2000)	*h* = −9→10
*T*_min_ = 0.859, *T*_max_ = 1.000	*k* = −17→9
8099 measured reflections	*l* = −17→17

### Refinement

**Table:**

Refinement on *F*^2^	Primary atom site location: structure-invariant direct methods
Least-squares matrix: full	Secondary atom site location: difference Fourier map
*R*[*F*^2^ > 2σ(*F*^2^)] = 0.052	Hydrogen site location: inferred from neighbouring sites
*wR*(*F*^2^) = 0.126	H-atom parameters constrained
*S* = 1.07	*w* = 1/[σ^2^(*F*_o_^2^) + (0.0257*P*)^2^ + 5.0797*P*] where *P* = (*F*_o_^2^ + 2*F*_c_^2^)/3
5309 reflections	(Δ/σ)_max_ < 0.001
262 parameters	Δρ_max_ = 1.69 e Å^−^^3^
0 restraints	Δρ_min_ = −1.98 e Å^−^^3^

### Special details

**Table:**

Geometry. All e.s.d.'s (except the e.s.d. in the dihedral angle between two l.s. planes) are estimated using the full covariance matrix. The cell e.s.d.'s are taken into account individually in the estimation of e.s.d.'s in distances, angles and torsion angles; correlations between e.s.d.'s in cell parameters are only used when they are defined by crystal symmetry. An approximate (isotropic) treatment of cell e.s.d.'s is used for estimating e.s.d.'s involving l.s. planes.
Refinement. Refinement of *F*^2^ against ALL reflections. The weighted *R*-factor *wR* and goodness of fit *S* are based on *F*^2^, conventional *R*-factors *R* are based on *F*, with *F* set to zero for negative *F*^2^. The threshold expression of *F*^2^ > σ(*F*^2^) is used only for calculating *R*-factors(gt) *etc*. and is not relevant to the choice of reflections for refinement. *R*-factors based on *F*^2^ are statistically about twice as large as those based on *F*, and *R*- factors based on ALL data will be even larger.

### Fractional atomic coordinates and isotropic or equivalent isotropic displacement parameters (Å^2^)

**Table:**

	*x*	*y*	*z*	*U*_iso_*/*U*_eq_	
Mn1	0.00869 (15)	0.35516 (10)	0.67962 (10)	0.0291 (3)	
I1	−0.28559 (8)	0.41022 (5)	0.57919 (5)	0.04330 (18)	
O1	−0.0292 (8)	0.4141 (5)	0.8476 (5)	0.0409 (14)	
H1A	−0.0480	0.4795	0.8793	0.061*	
H1B	0.0151	0.3979	0.9017	0.061*	
N1	0.0377 (8)	0.2491 (5)	0.5019 (5)	0.0241 (13)	
N2	−0.0908 (9)	0.0587 (6)	0.3252 (6)	0.0351 (16)	
N3	−0.2538 (9)	−0.0424 (6)	0.4477 (6)	0.0341 (16)	
N4	−0.1572 (8)	0.1567 (5)	0.6127 (5)	0.0280 (14)	
N5	0.2578 (8)	0.5264 (5)	0.7448 (6)	0.0290 (14)	
N6	0.5769 (9)	0.6325 (6)	0.8644 (6)	0.0318 (15)	
N7	0.5861 (9)	0.4338 (6)	0.8923 (6)	0.0353 (16)	
N8	0.2658 (8)	0.3303 (6)	0.7736 (5)	0.0297 (15)	
C1	0.1275 (11)	0.2974 (7)	0.4444 (7)	0.037 (2)	
H1	0.2053	0.3808	0.4861	0.045*	
C2	0.1111 (12)	0.2304 (8)	0.3274 (8)	0.042 (2)	
H2	0.1755	0.2659	0.2880	0.050*	
C3	−0.0021 (13)	0.1100 (9)	0.2699 (8)	0.043 (2)	
H3	−0.0179	0.0619	0.1885	0.052*	
C4	−0.0672 (10)	0.1311 (7)	0.4396 (7)	0.0284 (17)	
C5	−0.1668 (9)	0.0769 (6)	0.5026 (6)	0.0253 (16)	
C6	−0.3435 (11)	−0.0869 (8)	0.5083 (7)	0.037 (2)	
H6	−0.4092	−0.1721	0.4718	0.045*	
C7	−0.3448 (11)	−0.0163 (8)	0.6187 (8)	0.038 (2)	
H7	−0.4096	−0.0500	0.6592	0.046*	
C8	−0.2460 (10)	0.1078 (8)	0.6692 (7)	0.0341 (19)	
H8	−0.2416	0.1596	0.7469	0.041*	
C9	0.2540 (11)	0.6266 (8)	0.7371 (7)	0.038 (2)	
H9	0.1418	0.6243	0.6906	0.046*	
C10	0.4058 (11)	0.7330 (7)	0.7937 (8)	0.040 (2)	
H10	0.3993	0.8046	0.7900	0.048*	
C11	0.5674 (11)	0.7309 (7)	0.8557 (7)	0.038 (2)	
H11	0.6759	0.8022	0.8937	0.046*	
C12	0.4217 (10)	0.5351 (7)	0.8106 (6)	0.0277 (17)	
C13	0.4269 (10)	0.4261 (7)	0.8252 (6)	0.0290 (17)	
C14	0.5846 (11)	0.3351 (8)	0.9053 (7)	0.037 (2)	
H14	0.6966	0.3361	0.9507	0.045*	
C15	0.4283 (11)	0.2325 (8)	0.8558 (7)	0.0361 (19)	
H15	0.4284	0.1639	0.8675	0.043*	
C16	0.2732 (12)	0.2347 (7)	0.7892 (6)	0.0332 (19)	
H16	0.1638	0.1640	0.7517	0.040*	
I2	−0.22530 (8)	0.08320 (5)	0.95698 (5)	0.04477 (18)	
O2	0.0658 (7)	0.3656 (5)	1.0267 (5)	0.0432 (15)	
H2A	0.0086	0.2956	1.0159	0.065*	
H2B	0.1780	0.3826	1.0627	0.065*	

### Atomic displacement parameters (Å^2^)

**Table:**

	*U*^11^	*U*^22^	*U*^33^	*U*^12^	*U*^13^	*U*^23^
Mn1	0.0289 (6)	0.0251 (7)	0.0261 (6)	0.0058 (5)	0.0067 (5)	0.0086 (5)
I1	0.0360 (3)	0.0390 (4)	0.0546 (4)	0.0107 (3)	0.0067 (3)	0.0274 (3)
O1	0.054 (4)	0.046 (4)	0.031 (3)	0.028 (3)	0.017 (3)	0.020 (3)
N1	0.031 (3)	0.018 (3)	0.027 (3)	0.012 (3)	0.010 (3)	0.013 (3)
N2	0.042 (4)	0.028 (4)	0.031 (4)	0.010 (3)	0.013 (3)	0.010 (3)
N3	0.033 (4)	0.027 (4)	0.041 (4)	0.011 (3)	0.011 (3)	0.016 (3)
N4	0.032 (3)	0.022 (3)	0.025 (3)	0.004 (3)	0.008 (3)	0.010 (3)
N5	0.028 (3)	0.018 (3)	0.037 (4)	0.007 (3)	0.008 (3)	0.012 (3)
N6	0.033 (4)	0.022 (3)	0.037 (4)	0.007 (3)	0.013 (3)	0.011 (3)
N7	0.030 (4)	0.037 (4)	0.032 (4)	0.010 (3)	0.005 (3)	0.013 (3)
N8	0.029 (3)	0.026 (4)	0.027 (3)	0.010 (3)	0.002 (3)	0.008 (3)
C1	0.044 (5)	0.019 (4)	0.045 (5)	0.005 (4)	0.015 (4)	0.016 (4)
C2	0.053 (6)	0.041 (5)	0.043 (5)	0.016 (5)	0.027 (5)	0.026 (5)
C3	0.059 (6)	0.050 (6)	0.029 (5)	0.025 (5)	0.018 (4)	0.022 (4)
C4	0.031 (4)	0.022 (4)	0.031 (4)	0.008 (3)	0.009 (3)	0.012 (3)
C5	0.023 (4)	0.017 (4)	0.033 (4)	0.005 (3)	0.001 (3)	0.014 (3)
C6	0.029 (4)	0.030 (5)	0.040 (5)	0.004 (4)	0.001 (4)	0.014 (4)
C7	0.030 (4)	0.039 (5)	0.046 (5)	0.001 (4)	0.010 (4)	0.028 (4)
C8	0.034 (4)	0.046 (5)	0.036 (5)	0.024 (4)	0.017 (4)	0.024 (4)
C9	0.033 (4)	0.035 (5)	0.044 (5)	0.008 (4)	0.006 (4)	0.021 (4)
C10	0.043 (5)	0.028 (5)	0.056 (6)	0.014 (4)	0.015 (4)	0.027 (4)
C11	0.034 (4)	0.022 (4)	0.041 (5)	−0.004 (4)	0.010 (4)	0.008 (4)
C12	0.028 (4)	0.028 (4)	0.026 (4)	0.011 (3)	0.009 (3)	0.011 (3)
C13	0.028 (4)	0.022 (4)	0.022 (4)	0.000 (3)	0.006 (3)	0.003 (3)
C14	0.036 (5)	0.038 (5)	0.042 (5)	0.017 (4)	0.010 (4)	0.022 (4)
C15	0.041 (5)	0.031 (5)	0.039 (5)	0.018 (4)	0.016 (4)	0.015 (4)
C16	0.046 (5)	0.025 (4)	0.019 (4)	0.016 (4)	0.005 (4)	0.003 (3)
I2	0.0522 (4)	0.0349 (3)	0.0454 (4)	0.0132 (3)	0.0127 (3)	0.0203 (3)
O2	0.034 (3)	0.041 (4)	0.051 (4)	0.012 (3)	0.005 (3)	0.024 (3)

### Geometric parameters (Å, °)

**Table:**

Mn1—O1	2.115 (5)	C1—C2	1.374 (11)
Mn1—N1	2.256 (6)	C1—H1	0.9500
Mn1—N4	2.262 (6)	C2—C3	1.372 (12)
Mn1—N5	2.270 (6)	C2—H2	0.9500
Mn1—N8	2.304 (6)	C3—H3	0.9500
Mn1—I1	2.8048 (13)	C4—C5	1.486 (10)
O1—H1A	0.8400	C6—C7	1.361 (11)
O1—H1B	0.8400	C6—H6	0.9500
N1—C4	1.334 (9)	C7—C8	1.391 (11)
N1—C1	1.343 (9)	C7—H7	0.9500
N2—C3	1.338 (10)	C8—H8	0.9500
N2—C4	1.345 (9)	C9—C10	1.375 (11)
N3—C5	1.322 (9)	C9—H9	0.9500
N3—C6	1.350 (10)	C10—C11	1.374 (11)
N4—C8	1.332 (9)	C10—H10	0.9500
N4—C5	1.361 (9)	C11—H11	0.9500
N5—C9	1.333 (10)	C12—C13	1.492 (11)
N5—C12	1.348 (9)	C14—C15	1.373 (11)
N6—C11	1.328 (10)	C14—H14	0.9500
N6—C12	1.328 (9)	C15—C16	1.359 (11)
N7—C13	1.330 (9)	C15—H15	0.9500
N7—C14	1.338 (10)	C16—H16	0.9500
N8—C16	1.331 (10)	O2—H2A	0.8400
N8—C13	1.347 (9)	O2—H2B	0.8400
			
O1—Mn1—N1	166.1 (2)	C2—C3—H3	119.1
O1—Mn1—N4	94.6 (2)	N1—C4—N2	125.4 (7)
N1—Mn1—N4	72.8 (2)	N1—C4—C5	116.6 (6)
O1—Mn1—N5	93.0 (2)	N2—C4—C5	118.0 (7)
N1—Mn1—N5	97.0 (2)	N3—C5—N4	126.6 (7)
N4—Mn1—N5	159.3 (2)	N3—C5—C4	117.6 (7)
O1—Mn1—N8	84.1 (2)	N4—C5—C4	115.8 (6)
N1—Mn1—N8	89.6 (2)	N3—C6—C7	123.6 (8)
N4—Mn1—N8	89.4 (2)	N3—C6—H6	118.2
N5—Mn1—N8	72.2 (2)	C7—C6—H6	118.2
O1—Mn1—I1	93.91 (15)	C6—C7—C8	116.4 (8)
N1—Mn1—I1	93.41 (15)	C6—C7—H7	121.8
N4—Mn1—I1	96.02 (16)	C8—C7—H7	121.8
N5—Mn1—I1	102.63 (16)	N4—C8—C7	122.5 (7)
N8—Mn1—I1	174.35 (16)	N4—C8—H8	118.7
Mn1—O1—H1A	120.6	C7—C8—H8	118.7
Mn1—O1—H1B	125.9	N5—C9—C10	122.7 (8)
H1A—O1—H1B	108.5	N5—C9—H9	118.7
C4—N1—C1	116.3 (6)	C10—C9—H9	118.7
C4—N1—Mn1	117.2 (5)	C11—C10—C9	116.8 (7)
C1—N1—Mn1	125.6 (5)	C11—C10—H10	121.6
C3—N2—C4	116.9 (7)	C9—C10—H10	121.6
C5—N3—C6	115.3 (7)	N6—C11—C10	122.2 (7)
C8—N4—C5	115.6 (6)	N6—C11—H11	118.9
C8—N4—Mn1	127.6 (5)	C10—C11—H11	118.9
C5—N4—Mn1	116.8 (5)	N6—C12—N5	125.6 (7)
C9—N5—C12	115.7 (7)	N6—C12—C13	117.7 (7)
C9—N5—Mn1	126.1 (5)	N5—C12—C13	116.7 (7)
C12—N5—Mn1	117.6 (5)	N7—C13—N8	125.8 (7)
C11—N6—C12	116.9 (7)	N7—C13—C12	117.6 (7)
C13—N7—C14	116.0 (7)	N8—C13—C12	116.4 (7)
C16—N8—C13	115.4 (7)	N7—C14—C15	122.8 (8)
C16—N8—Mn1	127.8 (5)	N7—C14—H14	118.6
C13—N8—Mn1	116.7 (5)	C15—C14—H14	118.6
N1—C1—C2	122.4 (7)	C16—C15—C14	116.2 (8)
N1—C1—H1	118.8	C16—C15—H15	121.9
C2—C1—H1	118.8	C14—C15—H15	121.9
C3—C2—C1	117.3 (8)	N8—C16—C15	123.8 (8)
C3—C2—H2	121.4	N8—C16—H16	118.1
C1—C2—H2	121.4	C15—C16—H16	118.1
N2—C3—C2	121.8 (8)	H2A—O2—H2B	106.3
N2—C3—H3	119.1		
			
O1—Mn1—N1—C4	33.9 (12)	C1—N1—C4—C5	179.9 (6)
N4—Mn1—N1—C4	7.6 (5)	Mn1—N1—C4—C5	−10.5 (8)
N5—Mn1—N1—C4	169.2 (5)	C3—N2—C4—N1	−0.3 (12)
N8—Mn1—N1—C4	97.2 (5)	C3—N2—C4—C5	178.9 (7)
I1—Mn1—N1—C4	−87.6 (5)	C6—N3—C5—N4	1.1 (11)
O1—Mn1—N1—C1	−157.6 (8)	C6—N3—C5—C4	−179.5 (6)
N4—Mn1—N1—C1	176.1 (6)	C8—N4—C5—N3	−0.3 (11)
N5—Mn1—N1—C1	−22.3 (6)	Mn1—N4—C5—N3	179.1 (6)
N8—Mn1—N1—C1	−94.3 (6)	C8—N4—C5—C4	−179.7 (6)
I1—Mn1—N1—C1	80.9 (6)	Mn1—N4—C5—C4	−0.4 (8)
O1—Mn1—N4—C8	1.8 (6)	N1—C4—C5—N3	−172.3 (6)
N1—Mn1—N4—C8	175.7 (7)	N2—C4—C5—N3	8.4 (10)
N5—Mn1—N4—C8	113.1 (8)	N1—C4—C5—N4	7.2 (9)
N8—Mn1—N4—C8	85.9 (6)	N2—C4—C5—N4	−172.1 (7)
I1—Mn1—N4—C8	−92.6 (6)	C5—N3—C6—C7	−0.8 (11)
O1—Mn1—N4—C5	−177.4 (5)	N3—C6—C7—C8	−0.3 (12)
N1—Mn1—N4—C5	−3.6 (5)	C5—N4—C8—C7	−1.0 (11)
N5—Mn1—N4—C5	−66.2 (9)	Mn1—N4—C8—C7	179.8 (6)
N8—Mn1—N4—C5	−93.4 (5)	C6—C7—C8—N4	1.3 (12)
I1—Mn1—N4—C5	88.2 (5)	C12—N5—C9—C10	−0.9 (12)
O1—Mn1—N5—C9	−93.1 (7)	Mn1—N5—C9—C10	170.3 (6)
N1—Mn1—N5—C9	96.7 (7)	N5—C9—C10—C11	2.7 (13)
N4—Mn1—N5—C9	155.4 (7)	C12—N6—C11—C10	−0.4 (12)
N8—Mn1—N5—C9	−176.0 (7)	C9—C10—C11—N6	−2.0 (13)
I1—Mn1—N5—C9	1.6 (7)	C11—N6—C12—N5	2.5 (11)
O1—Mn1—N5—C12	78.0 (5)	C11—N6—C12—C13	−176.6 (7)
N1—Mn1—N5—C12	−92.2 (5)	C9—N5—C12—N6	−1.8 (11)
N4—Mn1—N5—C12	−33.5 (10)	Mn1—N5—C12—N6	−173.8 (6)
N8—Mn1—N5—C12	−4.9 (5)	C9—N5—C12—C13	177.2 (7)
I1—Mn1—N5—C12	172.7 (5)	Mn1—N5—C12—C13	5.2 (8)
O1—Mn1—N8—C16	84.7 (6)	C14—N7—C13—N8	2.3 (11)
N1—Mn1—N8—C16	−82.8 (6)	C14—N7—C13—C12	178.7 (7)
N4—Mn1—N8—C16	−10.0 (6)	C16—N8—C13—N7	−2.6 (11)
N5—Mn1—N8—C16	179.8 (7)	Mn1—N8—C13—N7	173.7 (6)
O1—Mn1—N8—C13	−91.1 (5)	C16—N8—C13—C12	−179.1 (6)
N1—Mn1—N8—C13	101.4 (5)	Mn1—N8—C13—C12	−2.8 (8)
N4—Mn1—N8—C13	174.2 (5)	N6—C12—C13—N7	0.8 (10)
N5—Mn1—N8—C13	4.0 (5)	N5—C12—C13—N7	−178.4 (6)
C4—N1—C1—C2	1.0 (11)	N6—C12—C13—N8	177.5 (6)
Mn1—N1—C1—C2	−167.6 (6)	N5—C12—C13—N8	−1.6 (10)
N1—C1—C2—C3	−0.1 (13)	C13—N7—C14—C15	−1.8 (12)
C4—N2—C3—C2	1.4 (12)	N7—C14—C15—C16	1.6 (12)
C1—C2—C3—N2	−1.2 (13)	C13—N8—C16—C15	2.4 (11)
C1—N1—C4—N2	−0.8 (11)	Mn1—N8—C16—C15	−173.4 (6)
Mn1—N1—C4—N2	168.7 (6)	C14—C15—C16—N8	−2.0 (12)

### Hydrogen-bond geometry (Å, °)

**Table:**

*D*—H···*A*	*D*—H	H···*A*	*D*···*A*	*D*—H···*A*
O1—H1A···O2^i^	0.84	1.93	2.767 (8)	172.
O1—H1B···O2	0.84	1.82	2.645 (8)	167.
O2—H2A···I2	0.84	2.60	3.423 (6)	168.
O2—H2B···N6^ii^	0.84	2.06	2.871 (8)	162.
O2—H2B···N7^ii^	0.84	2.38	2.918 (9)	122.

Symmetry codes: (i) −*x*, −*y*+1, −*z*+2; (ii) −*x*+1, −*y*+1, −*z*+2.
